# The effect of brief versus individually tailored dietary advice on change in diet, lipids and blood pressure in patients with inflammatory joint disease

**DOI:** 10.29219/fnr.v62.1512

**Published:** 2018-09-04

**Authors:** Maria Grorud Fagerhøi, Silvia Rollefstad, Sissel Urke Olsen, Anne Grete Semb

**Affiliations:** 1 Department of Clinical Service, Diakonhjemmet Hospital, Oslo, Norway; 2 Preventive Cardio-Rheuma Clinic, Department of Rheumatology, Diakonhjemmet Hospital, Oslo, Norway

**Keywords:** nutrition, dietary advice, diet, cardiovascular disease, inflammatory joint disease, lipids, blood pressure

## Abstract

**Background:**

Patients with inflammatory joint diseases (IJD) have an increased risk of cardiovascular disease (CVD) compared to the general population. Nutritional advice has been shown to influence CVD risk factors. Our objective was to evaluate whether an individually tailored dietary counselling versus a brief standardised advice on heart-friendly diet had comparable effect on change in diet, lipids and blood pressure (BP) in patients with IJD.

**Methods:**

Thirty-one patients with IJD aged 40–80 years received a brief standardised advice (4 min) on heart-friendly diet by a physician. Sixteen of the patients were randomised to receive an additional, individually tailored, heart-friendly dietary counselling session (60 min) by a dietitian. Change in dietary habits, measured by a validated questionnaire (SmartDiet), lipids, BP and C-reactive protein (CRP) were assessed after 8 weeks of follow-up.

**Results:**

After 8 weeks, the average increase in SmartDiet score was 5.1 and 5.7 points in the diet group (DG) and the control group (CG), respectively ( *p* = 0.65). Low-density lipoprotein cholesterol (LDL-c) was reduced by 12.6% in the DG versus 2.4% in the CG ( *p* = 0.05). There were no significant differences between the two groups regarding change in BP, lipids or CRP.

**Conclusion:**

Individually tailored dietary counselling resulted in more heart-friendly food choices in patients with IJD. However, the change in SmartDiet score was comparable for IJD patients receiving a brief nutritional advice and individually tailored heart-friendly dietary counselling. Further studies evaluating the longitudinal effects of dietary advice on CVD outcome in patients with IJD are warranted.

Cardiovascular disease (CVD) is the major cause of mortality worldwide, accountable for approximately 17.5 million deaths per year, representing 31% of all global mortality (1). Despite the fact that CVD mortality has decreased since the 1970s, it still remains as the leading cause of death in Norway (2). It is well established that patients with inflammatory joint diseases (IJD), such as rheumatoid arthritis (RA), psoriatic arthritis (PsA) and ankylosing spondylitis (AS), have an increased risk of atherosclerotic CVD compared to the general population (3, 4, 5, 6, 7). Several CVD risk factors are present in patients with IJD, including chronic inflammation, which may contribute to the increased CVD risk in this group. In addition, patients with RA have 2–3 times more asymptomatic atherosclerotic plaques in the carotid arteries compared to the general population (8). Thus, RA patients have an increased risk of myocardial infarction and sudden death (6, 9, 10). Hyperlipidaemia is prevalent in RA patients and has been reported to be present in 55–60% of the patients (11). In a meta-analysis comparing RA patients with and without hypercholesterolaemia, a 73% increased risk of CVD morbidity was reported in RA patients with increased lipid levels (12).

The role of food in the management of RA is controversial, and despite this, RA patients have been reported to regard food to be of importance in relation to their symptom severity and have been willing to change diet in an attempt to decrease their symptoms (13). Different hypotheses regarding the importance of diet in IJD patients have been proposed, which indicates that diet and lifestyle may play a role in both the development of and the course of the rheumatic disease (14, 15). Laboratory animal studies suggest that diet may have an impact on disease activity in IJD patients, although human studies are still scarce (16). Various dietary patterns, interventions and nutrients have been tested over the past decades (16, 17). The potential effect of this is still questionable. Today, effective anti-rheumatic treatment (synthetic disease-modifying anti-rheumatic drugs [sDMARDs]/biologic disease-modifying anti-rheumatic drugs [bDMARDs]) exists (16), which may reduce the importance of diet as a potential contributor to aggravation of disease activity. Nevertheless, diet will still be of considerable importance related to other aspects of IJD. Ensuring adequate and proper nutrition may be essential for further prognosis and in the prevention of comorbidities. The increased risk of CVD in IJD patients makes the prevention of comorbidities of especially importance. Modification of lifestyle-related risk factors, such as diet, is important in CVD prevention (18, 19, 20, 21, 22). Nutritional advice has been reported to influence CVD risk factors (18, 23).

SmartDiet, a validated questionnaire developed by the Lipid Clinic at Oslo University Hospital, has been shown to efficiently provide good estimates of diet and lifestyle habits in clinical practice (24). SmartDiet includes both qualitative and quantitative questions about average use of different food groups and beverages.

Whether the impact of traditional CVD risk factors on CVD morbidity in IJD patients diverges from that of the general population remains unknown (4, 12, 25). Furthermore, there is a knowledge gap regarding the effect of nutritional advice on change in dietary habits and CVD risk factors in IJD patients. The objective of this article was to evaluate whether an individually tailored heart-friendly dietary counselling by a dietitian and a standardised brief advice on heart-friendly diet given by a physician had comparable effects on change in diet, lipids and blood pressure (BP).

## Methods and materials

### Patients

Patients with IJD from the rheumatology outpatient clinic at Diakonhjemmet Hospital, or from primary care physicians, were referred to the Preventive Cardio-Rheuma Clinic, Department of Rheumatology, Diakonhjemmet Hospital, between January and June 2016. Inclusion criteria were as follows: patients must have RA, PsA or AS; be under the age group of 40–80 years; should be statin-naïve and have an indication for statin therapy as a primary or secondary prevention of CVD. Exclusion criteria were the following: 1) already diagnosed atherosclerotic CVD as previous myocardial infarction, coronary intervention (coronary artery bypass grafting or percutaneous coronary intervention), transient ischaemic attack/ischaemic stroke, stenosis of the carotid artery >50% and/or symptomatic carotid artery atherosclerosis; 2) BP >160/100 mmHg and/or medically treated hypertension; or 3) indications of familial hypercholesterolaemia (total cholesterol [TC] >7.5 mmol/L and low-density lipoprotein cholesterol [LDL-c] >4.9 mmol/L).

**Fig. 1:**
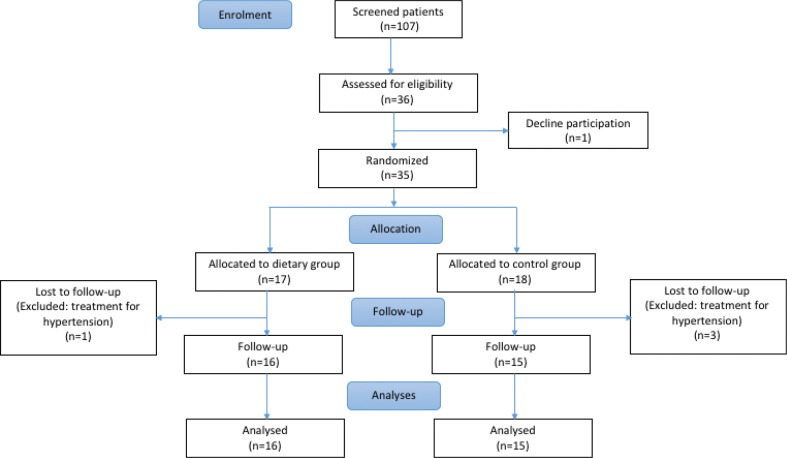
Flowchart of study design.

This study was an open randomised controlled clinical trial (RCT) with two treatment groups: a diet group (DG) and a control group (CG) (Fig. 1). A statistician developed the computer-generated randomisation list. Two independent secretaries compiled randomisation envelopes, which was based on the randomisation list. Inside the envelope a sheet describing the allocated treatment group was inserted into a dyed sheet, to further ascertain that it was not possible to reveal the treatment group without opening the envelope. Patients in the study were assigned randomisation numbers sequentially. Randomisation number and treatment group were recorded in each Case Report File.

All patients at the Cardio-Rheuma Clinic answered a questionnaire encompassing smoking status, presence of diabetes mellitus, medication history, family history of premature CVD in first-degree relatives, presence of established CVD, history of stroke, transient ischaemic attack and peripheral vascular disease. All the participants followed a standard procedure for CVD risk evaluation, which has previously been described in detail (26). Blood samples were drawn for laboratory tests including lipid profile, C-reactive protein (CRP), erythrocyte sedimentation rate, renal and liver function tests. Blood pressure was measured. The SmartDiet questionnaire was administered to all the patients by a dietitian. The data collection fulfilled the conditions of privacy and information security according to the Declaration of Helsinki (27). The study was approved by the South East Health Authority Ethical Committee for Medical Research (No. 2015/2087), and all the patients gave written informed consent to participate in the study.

### Dietary counselling

#### Brief standardised advice

The standardised dietary advice was given by an experienced cardiologist (Anne Grete Semb) who has used this set-up for several years at the Preventive Cardio-Rheuma Clinic. A list of five standardised clauses related to heart-friendly food was given along with a brochure including heart-friendly food items, which was developed by dietitians at Diakonhjemmet Hospital. Furthermore, due to time restriction during the consultation, the patients were informed that a detailed discussion about the patients’ diet could not be possible. This brief advice has been fixed to take 4 min. The standardised advice and the brochure have therefore been the same for patients in both study groups. The study also validated the use of the brochure together with the standardised brief advice as information towards change in food habits.

#### Individual tailored diet counselling

Participants in the DG received an individually tailored heart-friendly dietary advice during a session of 60 min, which was given by a dietitian. The information provided was based on the answers from the SmartDiet questionnaire. Main topics were discussed with all the participants, emphasising the importance of replacing saturated fat from full-fat dairy products, animal products, snacks, pastries and chocolate with unsaturated fat from marine sources, such as oily fish, and vegetable sources including oils, nuts, almonds, seeds and avocado (6, 28, 29, 30, 31, 32, 33).

Furthermore, to increase the intake of fiber through wholegrain cereals, especially oat (34, 35), fruits, berries and vegetables including legumes (36, 37), information on how to read food labels was also addressed. The importance of salt reduction (38, 39) was emphasised to all the participants, and alcohol consumption was discussed thoroughly if the patient had a high alcohol consumption and/or hypertriglyceridaemia. Coffee habits were addressed if frequently use of unfavourable brewing methods were reported (40). Portion control was not addressed specifically; however, patients with a high body mass index (BMI) asked for personal guidance regarding weight loss.

The participants were encouraged not to take other supplements than cod liver oil or similar omega-3 supplements if they were already taking such when entering the trial, and they were not asked to stop taking them. However, participants taking supplements other than cod liver oil or omega-3 supplements were not excluded from the trial.

### SmartDiet

The third version of the SmartDiet questionnaire (24) was handed out and collected by the dietitian in both groups at baseline and after 8 weeks of follow-up. SmartDiet has been developed in Norway and was designed to fit the Norwegian food habits. The questionnaire is not designed to measure the quantity of food, rather the frequency of food consumption and habits; therefore, portion size was not known. On the other hand, the questionnaire offers an opportunity to discuss central points of the patient’s dietary habits, and compliance was assessed by using the answers at 8 weeks follow-up. The questionnaire contains 15 point scoring questions, and it is possible to obtain a score between 15 and 45 points. An increase in score of at least 3 points from baseline to 8 weeks indicated a clinically significant improvement of the diet (24). A low score (≤ 27) indicates that improvement in many areas is needed to obtain a more heart-friendly diet. A medium score (28–35 points) indicates that the diet still needs improvement, while a high score (≥ 36 points) implies that the participants have healthy dietary habits. In the following, some sample questions from the SmartDiet questionnaire are presented. Each question has three or four response categories. Luncheon meat: what kind of luncheon meat do you use most often in your sandwich? A list of high- and low-fat luncheon is provided in addition to a category of consuming luncheon meats less than once a week, or never. Another sample question was about fish for dinner: how many times do you eat a fish product, and how many of those include fatty fish?

### CVD risk factors

The physician performed measurement of waist circumference, BP and a 12-lead electrocardiogram (ECG). Smoking status, physical activity habits, comorbidities, medication use and family history of CVD were also recorded. Body weight was measured by the dietitian at both baseline and at 8 weeks.

The blood samples were drawn in relation to the consultation with the physician, and the laboratory tests included TC, high-density lipoprotein cholesterol (HDL-c), triglycerides (TG) and CRP, which were measured at the hospital laboratory by routine procedures using COBAS 6000 modular and COBAS 8000 modular delivered by Roche Diagnostics Norge AS. LDL-c was calculated according to Friedewald’s formula (41).

Brachial BP was measured three times if it was found to be > 140/90 mmHg, using an Omron^®^ 7 series and Welch Allyn^®^ ProBP 3400 Series, by the physician, after 5 min rest in a supine position. A mean of the two last measurements was calculated. At 8 weeks follow-up, the dietitian performed the waist circumference and BP measurements.

### Statistics

Demographic characteristics of patients with IJD are presented as crude data, and the results are expressed as mean ± standard deviation (SD), or median and interquartile range (IQR) for normally and non-normally distributed continuous variables, respectively. Categorical variables are presented as numbers and percentages (%). Variables with a normal distribution were analysed using independent samples t-test and analysis of co-variance (ANCOVA), for group comparisons, with baseline values as covariates. A supplementary model including baseline values, BMI, systolic BP (SBP) and diastolic BP (DBP) as covariates was conducted. Non-normally distributed variables (TG and CRP) were log-transformed before comparison. For skewed continuous variables (HDL-c), the non-parametric Mann–Whitney U test was conducted to compare per cent change from baseline to follow-up. The primary analyses followed the intention-to-treat principle. Missing data were handled using pairwise deletion. The sample size calculations were performed by a statistician. With an estimated difference between the groups of three SmartDiet points, and an SD of 2.69 for change from baseline to follow-up, at least 13 patients were calculated to be needed in each group to show a statistical difference (two-sided t-test, 5% significance level) between the groups at 80% strength. The corresponding count for 90% strength was 17 patients completed in each group. The level of statistical significance was set at a *p* ≤0.05 for all analyses. Statistical Package for the Social Sciences (SPSS) version 23 was used for the statistical analyses.

## Results

### Patient characteristics

Baseline characteristics of the study participants are shown in Table 1. Thirty-one patients with IJD (RA, *n* = 16; PsA, *n* = 7; and AS, *n* = 8) were included in the study, of whom 16 were randomised to the DG. Females dominated in both groups (DG: 56.3%, CG: 60.0%) ( *p* = 0.83). The participants in the DG had lower BMI ( *p* = 0.03) and waist circumference ( *p* = 0.002) compared to those in the CG. The lipid profile was comparable, and median CRP was < 5 mg/L in both groups. SBP ( *p* = 0.04) and DBP ( *p* = 0.04) were higher among patients in the CG compared to those in the DG. There were no significant differences in medication use between the groups. Among the patients in the DG, 81.3% were using bDMARDs compared to 53.3% of the patients in the CG ( *p* = 0.14), while 43.8 and 60.0% of the patients in both groups used sDMARDs ( *p* = 0.37), respectively. Current prednisolone medication use was present in 6.3 and 20.0% among the DG and CG patients, respectively ( *p* = 0.33).

**Table 1:** Baseline characteristics of all patients, diet group and control group

Baseline characteristics	All patients, *n* = 31	Diet group, *n* = 16	Control group, *n* = 15	*p*-value^a^
Diagnosis RA/PsA/AS n (%)	16 (51.6)/7 (22.6)/8 (25.8)	7 (43.8)/4 (25.0)/5 (31.3)	9 (60.0)/3 (20.0)/3 (20.0)	0.72^c^
Sex male/female n (%)	13(41.9)/18 (58.1)	7 (43.8)/9 (56.3)	6 (40.0)/9 (60.0)	0.83^b^
Age mean ± SD	54.94 ± 9.96	53.38 ± 10.36	56.60 ± 8.91	0.36
**Lipids**				
TC (mmol/L) mean ± SD	5.88 ± 0.84	6.10 ± 0.85	5.64 ± 0.78	0.13
HDL-c (mmol/L) mean ± SD	1.48 ± 0.42	1.46 ± 0.47	1.50 ± 0.38	0.82
LDL-c (mmol/L) mean ± SD	3.71 ± 0.83	3.90 ± 0.95	3.52 ± 0.66	0.21
TG (mmol/L) median (IQR)	1.29 (1.08)	1.29 (0.98)	1.29 (1.23)	0.91
**Blood pressure, mean ± SD**				
Systolic (mmHg)	129.06 ± 17.11	122.96 ± 14.30	135.57 ± 17.89	**0.04**
Diastolic (mmHg)	80.85 ± 9.80	77.34 ± 10.16	84.60 ± 8.14	**0.04**
**Inflammation biomarkers, median (IQR)**				
CRP (mg/L)	2.00 (4.00)	2.00 (4.00)	3.50 (5.25)	0.15
Sedimentation rate (mm)	17.00 (16)	16.50 (16)	17 (14)	0.72
**Antropomethry, mean ± SD**				
Weight (kg)	81.04 ± 12.26	76.43 ± 15.89	85.95 ± 13.35	0.08
BMI (kg/m2)	27.56 ± 4.89	25.76 ± 1.19	29.49 ± 1.13	**0.03**
Waist circumference (cm)	96.48 ± 11.44	90.69 ± 9.94	102.67 ± 9.78	**0.002**
**Smoking habits, n (%)**				
Daily smoking	7 (22.6)	3 (18.8)	4 (26.7)	0.69^c^
Social smoking	2 (6.5)	2 (12.5)	0 (0.0)	0.48^c^
**Comorbidities, n (%)**				
Combined dyslipidaemia^d^	3 (9.7)	1 (6.3)	2 (14.3)	0.59^c^
Hyperlipidaemia^e^	14 (45.2)	9 (56.3)	5 (33.3)	0.20^b^
Hypertension^f^	8 (25.81)	2 (12.6)	6 (40.0)	0.15^b^
Diabetes	0 (0.0)	0 (0.0)	0 (0.0)	-
Carotid plaque	26 (83.9)	13 (81.3)	13 (86.7)	1.0^c^
**Medication, n (%)**				
Prednisolone	4 (12.9)	1 (6.3)	3 (20.0)	0.33^c^
NSAIDs	12 (38.7)	6 (37.5)	6 (40.0)	0.89^b^
sDMARDs	16 (51.6)	7 (43.8)	9 (60.0)	0.37^b^
bDMARDs	21 (67.7)	13 (81.3)	8 (53.3)	0.14^c^

a Differences between the diet group and the control group at baseline, analysed by independent samples t-test.

b Pearson’s chi-square test,

c Fisher’s exact test

d Triglycerides > 2 mmol/L, HDL < 1 mmol/L

e Total-cholesterol > 6 mmol/L

f Systolic blood pressure > 140 mmol/L

RA, rheumatoid arthritis; PsA, psoriatic arthritis; AS, ankylosing spondylitis; SD, standard deviation; TC, total cholesterol; HDL-c, high-density lipoprotein cholesterol; LDL-c, low-density lipoprotein cholesterol; TG, triglycerides; IQR, interquartile range; BMI, body mass index; CRP, C-reactive protein; NSAIDS, non-steroidal anti-inflammatory drugs; sDMARDs, synthetic disease-modifying anti-rheumatic drugs; bDMARDs, biologic disease-modifying anti-rheumatic drugs.

Bold values: significant values

### Diet

There were no significant differences in change in SmartDiet score from baseline to after 8 weeks follow-up between the DG and the CG ( *p* = 0.65), and no further change was observed after adjusting for baseline SmartDiet score, BMI and BP ( *p* = 0.26) (Table 2).

**Table 2:** Effect of extended and individually tailored nutritional consultation (diet group) on diet, lipoproteins, blood pressure and inflammatory markers versus standardised brief advice (control group)

Clinical endpoints	Diet group, *n* = 16		Control group, *n* = 15		Unadjusted mean group difference (95 % CI)^b^	*p*-value^a^	Estimated mean group difference (95 % CI)^b^	*p*-value^c^	Estimated mean group difference (95 % CI)^b^	*p*- value^d^
	
	Baseline	8 weeks	Baseline	8 weeks						
**Diet**										
SmartDiet sum score mean ± SD	28.25 ± 2.89	33.31 ± 2.73	25.60 ± 4.63	31.27 ± 5.08	-0.60 (-3.29, 2.09)	0.65	0.46 (-2.19, 3.10)	0.73	1.55 (-1.24, 4.34)	0.26
**Lipids**										
Total cholesterol (mmol/L), mean ± SD	6.10 ± 0.85	5.73 ± 1.12	5.64 ± 0.78	5.55 ± 0.58	-0.28 (-0.81, 0.25)	0.29	-0.15 (-0.69, 0.38)	0.56	-0.13 (-0.76, 0.51)	0.68
HDL-cholesterol (mmol/L), mean ± SD	1.46 ± 0.47	1.51 ± 0.54	1.50 ± 0.38	1.48 ± 0.28	0.07 (-0.13, 0.26)	0.50	0.06 (-0.13, 0.25)	0.53	0.05 (-0.19, 0.28)	0.70
LDL-cholesterol (mmol/L), mean ± SD	3.89 ± 0.95	3.42 ± 1.04	3.52 ± 0.66	3.36 ± 0.47	-0.31 (-0.71, 0.08)	0.11	-0.23 (-0.62, 0.16)	0.23	-0.26 (-0.71, 0.19)	0.25
Triglycerides (mmol/L), median (IQR)	1.29 (1.86, 0.88)	1.62 (2.08, 0.84)	1.29 (2.05, 0.82)	1.36 (1.91, 0.86)	1.06 (0.84, 1.35)	0.63	-0.06 (-0.18, 0.29)	0.62	0.06 (-0.23,0.35)	0.67
**Blood pressure**										
Systolic blood pressure (mmHg), mean ± SD	122.96 ± 14.30	121.81 ± 12.39	135.57 ± 17.89	130.03 ± 13.39	4.38 (-3.67, 12.43)	0.28	-0.80 (-7.84, 6.23)	0.82	0.07 (-7.20, 7.35)	0.98
Diastolic blood pressure (mmHg), mean ± SD	77.34 ± 10.16	77.78 ± 7.56	84.60 ± 8.14	82.87 ± 7.90	2.17 (-3.71, 8.05)	0.46	-1.65 (-6.79, 3.50)	0.52	-0.65 (-6.27, 4.97)	0.81
**Inflammation biomarker**										
C- reactive protein (mg/L), median (IQR)	2.00 (4.00, 0.00)	2.00 (4.75, 1.00)	3.50 (7.25, 2.00)	4.00 (8.00, 2.00)	1.28 (0.30, 79.45)	0.25	0.18 (-2.08, 2.43)	0.87	1.08 (-1.50, 3.67)	0.40

a Differences from pre- to post-intervention (8 weeks) values, between the groups, analysed with independent samples t-test.

b Estimated regressions coefficients.

c Estimated mean group difference values, analysed with ANCOVA, with baseline values as covariates.

d Estimated mean group difference values, analysed with ANCOVA, with baseline, BMI, SBP and DBP values as covariates.

SD, standard deviation; TC, total cholesterol; HDL-c, high-density lipoprotein cholesterol; LDL-c, low-density lipoprotein cholesterol; TG, triglycerides; IQR, interquartile range; BMI, body mass index; SBP, systolic blood pressure; DBP, diastolic blood pressure; CI confidence interval; ANCOVA, analysis of co-variance.

**Fig. 2:**
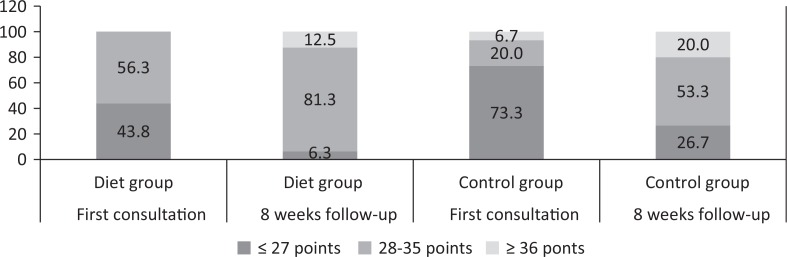
Distribution of score obtained by SmartDiet. Points equal to or less than 27 indicates that the diet should be improved in several areas, a score of 28–35 points indicates that the diet could still be improved to become more heart-friendly, while a score of ≥ 36 points indicates that the dietary habits are heart-friendly and healthy.

At baseline, 43.8% and 73.3% of the patients in the CG and the DG had a low SmartDiet score ( *p* = 0.95); on the other hand, more patients in the DG group had a medium SmartDiet score compared to the CG: 56.3% versus 20.0% ( *p* = 0.04), respectively (Fig. 2). After 8 weeks of follow-up, the SmartDiet score in the DG and CG was for: 1) low SmartDiet score: 6.3% versus 26.7% ( *p* = 0.17) and 2) medium SmartDiet score: 81.3% versus 53.3% ( *p* = 0.14) (Fig. 2). At least 3 points improvement in SmartDiet score was obtained by 87.5% in the DG ( *p* < 0.001) and 80.0% in the CG ( *p* < 0.001).

The increase in SmartDiet score from baseline to 8 weeks follow-up reflects several changes in dietary habits (supplementary Table 1). In summary, the changes towards a more heart-friendly diet were greatest among patients in the DG, who more frequently used vegetable oil/liquid margarine for cooking (93.8% vs. 60.0%) ( *p* = 0.04) and had a higher consumption of whole grain bread >6 grams of fibre/100g (93.8% vs. 60.0%) ( *p* = 0.04). On the other hand, patients in the CG reported frequently use of butter/hard margarine on bread compared to the DG (46.7% vs. 6.7%) ( *p* = 0.02).

### Lipids

**Fig. 3:**
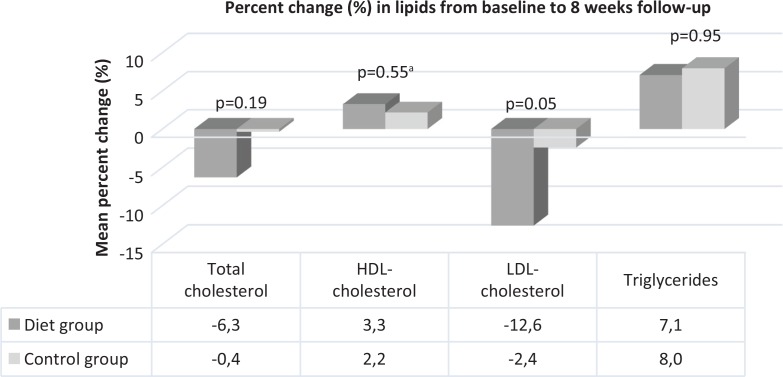
Per cent change in lipids from baseline to 8 weeks of follow-up in the diet group and control group. Differences between the diet group and the control group are analysed by an independent samples t-test. ^a^Mann–Whitney U test.

TC ( *p* = 0.13), LDL-c ( *p* = 0.21), HDL-c ( *p* = 0.82) and TG ( *p* = 0.91) were comparable in both DG and CG at baseline [Table 1]. Eight weeks after the individually tailored heart-friendly dietary counselling by a dietitian, no significant differences were found between the groups regarding TC ( *p* = 0.29), LDL-c ( *p* = 0.11), HDL-c ( *p* = 0.50) or TG ( *p* = 0.63) (Table 2). Adjustment for baseline values of BMI and BP did not change the outcome, nor did the adjustment of omega-3 usage influence TG levels in any of the groups. Figure 3 illustrates the per cent change in lipids from baseline to follow-up. Both groups had an average decrease in TC from the first to the final consultation, corresponding to −6.3% versus −0.4 % in the DG and the CG ( *p* = 0.19), respectively. A mean per cent decrease of −12.6 and −2.4% in LDL-c ( *p* = 0.05) and a mean per cent increase of 3.3 and 2.2% ( *p* = 0.55) in HDL-c in the DG and CG, respectively, were demonstrated. There was a comparable mean per cent increase in TG in both the groups (DG 7.1% vs. CG 8.0%) ( *p* = 0.95).

### Blood pressure

**Fig. 4:**
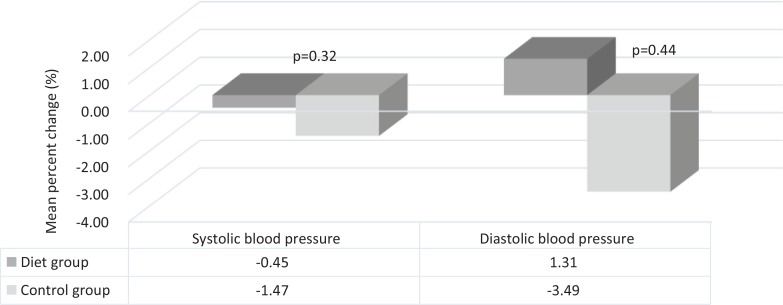
Per cent change in blood pressure from baseline to 8 weeks of follow-up in the diet group and control group. Differences between the diet group and the control group are analysed by an independent samples t-test.

No significant mean differences in SBP or DBP between the DG and the CG after 8 weeks follow-up were revealed ( *p* = 0.28, *p* = 0.46) (Table 2). Adjusting for baseline values of BMI and BP did not change the outcome (Table 2). Both groups showed a decline in SBP from baseline to 8 weeks follow-up (−1.15 mmHg vs. −5.53 mmHg in the DG and CG, respectively, corresponding to a per cent change in SBP of 0.45 and 1.47% ( *p* = 0.32) (Fig. 4). DBP was, on average, increased by 0.44 mmHg (1.31%) in the DG and decreased by –1.73 mmHg (3.49%) in the CG ( *p* = 0.44).

### Inflammatory markers

No differences were observed in change in CRP values from baseline to follow-up between the DG and the CG ( *p* = 0.25) (Table 2). Adjusting for baseline values of BMI and BP did not change the outcome. Median CRP was unaltered (2.0 mg/L) from the first to the final consultation in the DG, while a modest increase from median 3.5 mg/L to 4.0 mg/L was revealed in the CG after 8 weeks ( *p* = 0.75).

## Discussion

We have shown that a standardised brief advice combined with a nutritional purchase guide brochure may be sufficient to improve the dietary habits towards a heart-friendly diet in patients with IJD. This study successfully improved dietary habits defined by > 3 points change in the SmartDiet score. Importantly, even short sessions on heart-friendly diet advice enable patients to make changes with clinical effect. However, the nutritional advice provided by a dietitian resulted in significant better compliance to the heart-friendly diet after 8 weeks.

### Dietary counselling

Few studies have compared the effectiveness of an individually tailored heart-friendly dietary counselling by a dietitian versus brief advice provided by physicians (23). One study reported a 12% short-term reduction in LDL-c after counselling by a dietitian versus a 7% reduction after a physician consultation (23). However, the observed effect did not sustain after 1 year, indicating that the effects of diet change on LDL-c after advice by a dietitian or a physician are comparable at 1 year follow-up. Another recently published study investigated the effectiveness of a brief dietary intervention on CVD risk factors in patients with hyperlipidaemia (42). In that study, the patients received a tailored dietary counselling mainly based on the Mediterranean diet and the Portfolio diet (43), combined with a nutritional educational manual, regarding eating patterns. After 6 weeks, a significant reduction in energy-dense/nutrient-poor foods was demonstrated. These results support the effect of a brief and single dietary counselling.

Professor Ingar Hjermann was a pioneer in dietary research and prevention of CVD, and through The Oslo Diet and Antismoking study it was revealed that men receiving dietary advice, primarily on reducing saturated fatty acids (SFA) and increasing their intake of polyunsaturated fatty acids (PUFA), had an average of 13% greater decrease in TC compared to the CG (44). A 20-year follow-up of this study showed that men in the intervention group still had a more conscious approach to diet and lifestyle and ate less SFA and cholesterol and more PUFA than the CG (45). Consequently, the Oslo study demonstrated that making lifestyle interventions, such as diet counselling, provides evident and lifelong effects, suggesting that diet counselling may result in permanent change in dietary habits.

Although several lifestyle intervention programmes have shown to be effective, it requires great resources and may not be available in the majority of health care systems. There is a knowledge gap regarding the effectiveness of less intensive counselling in relation to the minimum of, and the duration of, sessions needed to obtain alteration in dietary habits and clinical effects on other CVD risk factors (46). However, we have through our study contributed to this knowledge gap regarding patients with IJD.

### Lipids

There were no differences in the mean change in TC, LDL-c or HDL-c after 8 weeks between the two groups in our study. However, the per cent reduction was numerically greater for TC and significantly higher for LDL-c in the DG, which favours individual tailored diet counselling.

Our findings are in accordance to previous studies (23, 43, 47), where heart-friendly dietary habits have demonstrated a reduction in LDL-c from 5 to 30% (43, 47). The differences observed in mean per cent change in LDL-c levels between the two groups in our study may be attributed to a more unfavourable choice of fat sources in the CG. Interestingly, a reduction of 1 mmol/L in LDL-c has been shown in large international, placebo controlled, randomised statin trials to be related to a risk reduction of future CVD of 21% (48). Promising results from *post hoc* analyses in two large statin trials found that both the lipid and risk reduction of future CVD in patients with IJD and non-IJD were comparable (49). Future prospective studies are warranted to answer whether LDL-c reduction due to dietary intervention has a clinical effect on CVD outcomes.

Results from a prospective cohort study revealed that replacing 5% of the energy intake from SFA with an equivalent energy intake from PUFAs or whole grain was associated with a 25 and 9% decrease in CVD risk, respectively (50).

Several lifestyle factors may influence TG levels, such as weight reduction, high consumption of refined carbohydrates (51), especially fructose (52) and/or high alcohol consumption (53). However, we did not observe any significant weight reduction in our study; rather, there were only minor changes of refined carbohydrates and beverages, including alcohol consumption, which cannot probably explain an increase in TG in either of the groups. Despite the increase in TG levels in our study, the mean levels were still < 1.7 mmol/L in both groups, which is the recommended level for TG (51).

### Blood pressure

We observed no significant differences in SBP or DBP after 8 weeks of follow-up between the DG and the CG. However, a clinical important reduction in SBP was observed in the CG (~5 mmHg), but not in the DG, and studies have shown that even a small reduction in SBP/DBP (~2 mmHg/ 1 mmHg) may cause a reduction in CVD morbidity and mortality (54).

A high consumption of sodium is a well-known risk factor for hypertension (55). Patients in the DG were recommended to decrease their salt intake. Unfortunately, SmartDiet does not give the opportunity to evaluate salt intake. However, in a recent review it was concluded that sodium modification in normotensive patients did not significantly alter BP (56). In our study, 87.4% of the patients in the DG were normotensive versus 60.0% in the CG. An increase in the consumption of fruit and vegetables is another dietary factor that has been associated with a reduction in BP (57, 58, 59), while excessive alcohol consumption is related to an increased risk of hypertension (60). There were no differences in fruit and vegetable intake or alcohol consumption between the groups after 8 weeks.

### Inflammatory markers

No significant changes in CRP levels were observed between the two groups after 8 weeks of follow-up. The majority of patients in this study were treated with potent anti-inflammatory medication, which may explain the absence of alterations in CRP levels. Previous studies have shown that dietary intervention may affect inflammation, but weight loss seems to be of more importance (61).

### Limitations

A limitation of our study is the collection of dietary information using a questionnaire. Recall bias will potentially lead to less accuracy on a patient’s dietary habits (62), while guidance by a dietitian during answering the questionnaire may have increased the risk of pleasing bias (62). Another limitation of the study is the difference in baseline data between the two groups. *A priori* calculations were performed to obtain 80% strength, for which a minimum of 13 patients was necessary in each group. Despite meeting the estimated minimum number of patients, there were significant differences between the groups at baseline, both in BMI and BP. The *a priori* power calculations were based on the difference between the groups, and not within each group. The changes in SmartDiet score within each group were comparable. There was a difference in baseline SmartDiet score, as well as differences in dietary quality between the groups at baseline. The change in diet quality was greatest among patients in the DG. However, both groups had major changes in their food habits during the study period.

SmartDiet score was used to evaluate changes in dietary habits of the participants, where an increase of at least 3 points was set as a basis to denote an improvement in diet (24). However, the 3-point score in the SmartDiet has never been assessed (24). Nevertheless, the questionnaire has been validated against a 7-day ‘weighed food consumption record’ (24) and was shown to provide good estimates of dietary fat and fiber intake, while the estimated intake of fish, vegetables and snacks was evaluated to be somewhat more imprecise. The lower sensitivity for evaluation of changes in the latter mentioned foods is a limitation of the SmartDiet method. The questionnaire has been used in several studies (63, 64), and also as a model in the development of a Canadian version of the questionnaire (65).

A further limitation of the study is the small sample size and the short-term follow-up. Studies have shown that to achieve long-lasting dietary alterations, it will require counselling on several occasions with an intensified dietary and lifestyle guidance (46, 66), and there is a need for studies testing a more intensive intervention with multiple sessions to be able to illuminate the long-term effect of change in dietary habits. However, this is the first study to evaluate the effect of dietary advice in patients with IJD.

## Conclusion

Our findings indicate that both standardised brief dietary advice and individually tailored dietary counselling lead to short-term improvement in heart-friendly diet evaluated by SmartDiet score in patients with IJD. Regarding health care resources, the positive result of a brief standardised advice may be of interest, although an individually tailored dietary counselling by a dietitian seems superior to brief advice regarding change to more heart-friendly food choices and reduction of LDL-c. Studies on long-term effects of dietary advice on CVD outcome in patients with IJD are warranted.
